# Intraspecific phenotypic differences in climbing perch *Anabas testudineus* (Bloch, 1792) populations may be linked to habitat adaptations

**DOI:** 10.1016/j.heliyon.2023.e17685

**Published:** 2023-06-28

**Authors:** Md. Rased Khan Manon, Asraful Alam, Md. Rahamat Ullah, Md. Belal Hossen, Md. Abu Sufian, Mohammad Amzad Hossain, Mohammed Mahbub Iqbal, Md. Arifur Rahman

**Affiliations:** a Patuakhali Science and Technology University, Patuakhali, 8602, Bangladesh; b Department of Fisheries Biology and Genetics, Patuakhali Science and Technology University, Patuakhali, 8602, Bangladesh; c Bangladesh Fisheries Research Institute, Riverine Sub-Station, Khepupara, Patuakhali, 8650, Bangladesh; d Department of Aquatic Resource Management, Sylhet Agricultural University, Sylhet, 3100, Bangladesh; e Department of Fish Biology and Genetics, Sylhet Agricultural University, Sylhet, 3100, Bangladesh

**Keywords:** *Anabas testudineus*, Condition factors, Morphometrics, Meristics, Conservation

## Abstract

The climbing perch, *Anabas testudineus,* is a nutritionally and economically significant food fish. The present study reveals the first comprehensive description of the life-history traits of *A. testudineus* scooped up through different traditional fishing gears from July 2020 to December 2020. Among the 120 collected specimens, the smallest and largest specimens were 8.5 cm–14.6 cm TL in Nilphamari and Patuakhali, respectively. The estimated b values for LLRs indicated positive allometric growth in all sampling points (b > 1.0). The LWRs of *A. testudineus* indicated positive allometric growth in the Gazipur and Nilphamari districts (b > 3.00) and negative allometric growth in the Patuakhali and Khulna districts (b < 3.00). A Wilcoxon sign-ranked test for *W*_*R*_ showed no significant dissimilarity from 100, signifying the balanced habitat for *A. testudineus.* The estimated *a*_*3.0*_ was minimum in Khulna (0.0110) and maximum in Nilphamari (0.0825). “The *Lm* was estimated at 7.4032 (7.4) cm TL in Nilphamari and 8.86 (8.9) cm TL in Patuakhali”. Nineteen of twenty morphometric measurements and ten of twelve meristic characters showed substantial variations (p < 0.0001). The principal component analysis indicated shape variation and explained 85.361% of the total variance and showed differences in TL, SL, HL, LBD, LE1, D1D2, A1A2, and VV2. The cluster heatmap demonstrates that the other stocks segregated Gazipur stock. Our findings reveal a significant dataset about intraspecific phenotypic differentiation, which will aid the long-term exploration and management of *A. testudineus* species in Bangladesh and its neighboring countries.

## 1 Introduction

The climbing perch, *Anabas testudineus* (Bloch, 1792) is distributed throughout Asia, including Bangladesh, and belongs to the Anabantidae family of the Perciformes order [1,2]. Even though adults prefer rivers, flooded fields, and slow-moving canals, it is a fresh and brackish water potamodromous species that primarily prefers canals, reservoirs, wetlands, swamps, and estuaries [3]. *A. testudineus* can tolerate challenging water conditions, such as little oxygen and contaminated water, because of an accessory air-breathing organ called the labyrinth organ [4]. This species is commercially important because it has a high market value in Southeast Asia as a delectable food fish [3]. It is also a native commercial target species and a vital source of income for small and large-scale fishermen who employ a range of traditional fishing techniques. In the 2019–20 fiscal year, the nation generated about 66497 metric tons of climbing perch. According to the Department of Fisheries Bangladesh, *Anabas testudineus* represents 1.74% of the nation's total inland fish production [5]. Even though this habitat-generalist fish faces potential concerns such as habitat degradation and drying out, it is classified as the “least concern” in Bangladesh [6].

Researchers frequently employ morphometric and meristic features in the discrimination and classification research of fish, and they frequently utilize statistical approaches [7,8]. The traditional ways of stock identification continue to play an important role in stock identification even today, despite the development of tools that particularly evaluate biochemical or molecular genetic variation [9]. In most cases, researchers use length-frequency distribution (LFD) studies to express the life-history traits and ecology of fish [10] and length-weight relationships (LWRs) for estimating weight, biomass, and condition indices [11]. Moreover, condition factors help in assessing the status of fish, allowing for the estimation of current and potential population success [[12], [13], [14]]. Furthermore, for the past two decades, relative weight (*W*_*R*_) has been one of the most widely known indices for evaluating fish conditions in the United States [14], and it is now being used in Bangladesh to determine freshwater fishes [[15], [16], [17]]. Several authors have reported on LWRs and the condition factors [16,18], morphometric and meristic variations [19], morphometric and gonadal studies [20], captive breeding [21], reproduction and spawning behavior [22], fecundity [23], induced breeding [24], and growth performance of *A. testudineus* [25]. However, researchers have not studied phenotypic variations linked to habitat adaptations based on a multi-model approach for this economically important species from Bangladesh or elsewhere. Thus, this study aimed to examine the phenotypic variations in the population of *A. testudineus* from different sources of Bangladeshi waters to recognize body shape plasticity and divergence using a multi-model approach based on morphometric and meristic characteristics.

## 2 Materials and methods

### 2.1 Sample collection

**Fig. 1:**
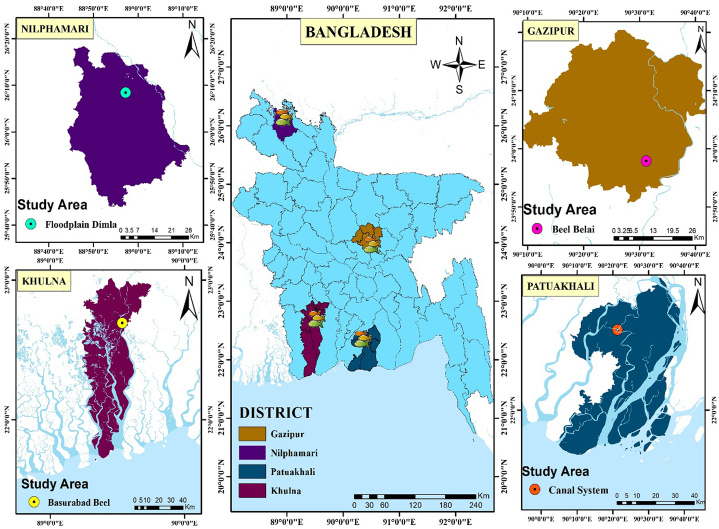
The map depicting the places of *A. testudineus* sample collection in Bangladesh.

During the study period of July 2020 to December 2020, a total of 120 fish were taken from Belai *beel* (Gazipur district), Floodplain Dimla (Nilphamari district), Canal system (Patuakhali district), and Basurabad *beel* (Khulna district), with 30 individuals from each region (Fig. 1). The traditional fishing gears such as gill nets (mesh sizes 1.0–3.0 cm), cast nets (mesh sizes 1.0–2.0 cm), square lift nets (mesh size ∼1.0 cm), and current jals (mesh sizes 1.0–2.0 cm) were used to collect the sample. All specimens were identified at the species level and the validity of the scientific name was confirmed in FishBase. Digital slide calipers (Mitutoyo, CD‐6″CSX, Japan) were used to measure the Total length (TL) and Standard length (SL) for each individual, and an electronic balance (AND, FSH, Korea) to measure entire body weight to the nearest 0.01 cm and 0.01 g precision, respectively.

### 2.2 Length-frequency distribution (LFD)

Length frequency distribution for this species considering the collection regions was constructed using a violin boxplot based on total length.

### 2.3 Length-length relationships (LLRs)

The length-length relationship with total length among different body lengths was determined by the method of least squares to fit a simple linear regression model as Y = a + bX, where Y = various body lengths, X = total length, a = proportionality constant, and b = regression coefficient.

### 2.4 Length-weight relationships (LWRs)

The length-weight relationship of the fish was estimated by the following equation of W = aL^b^ [26], where W = total weight of fish (g), L = total length of fish (mm), a = intercept (describe the rate of change of weight with length), b = slope (weight at unit length). The parabolic equation (*W = aL*^*b*^) was then transformed into a linear equation using a logarithmic method: Ln *W =* Ln *a + b* Ln L.

### 2.5 Condition factors

#### 2.5.1 Allometric condition factor (K_A_)

The element of allometric condition (*K*_*A*_) was calculated by the following equation of *K*_*A*_ = W/L^b^ [27], Where W = the body weight (g), L = the TL (cm), and b = the LWR parameter.

#### 2.5.2 Fulton's condition factor (K_F_)

Fulton's condition factor was determined by using the following equation of *K*_*F*_ = 100 × W/L^b^ [11,28]; Where *K*_*F*_ = condition factor, W = the weight of the fish (g), L = the total length of the fish in centimeters (cm), and b = the value obtained from the length-weight equation.

#### 2.5.3 Relative weight (W_R_)

The relative weight was calculated with the following formula: *W*_*R*_=(W/W_s_) × 100 [11], Where W = the weight of a particular individual, W_s_ = the predicted standard weight for the same individual, W_s_ as calculated by W_s_ = aL^b^ (a and b values obtained from the composite of length-weight relationships throughout the range of the species).

### 2.6 Form factor (a_3.0_)

The form factor (*a*_*3.0*_) of *A. testudineus* was estimated through the equation of: *a*_*3.0*_ = 10^log a – s (b−3)^ [11], where a and b are the regression parameters of LWR, and S is the regression slope of ln a vs. b. The researchers used a mean slope S = −1.358 for calculating the form factor because there was no available information on LWR for this species to estimate the regression (S) of ln a vs*.* b.

### 2.7 Size at first sexual maturity (L_m_)

The size of *A. testudineus* at first sexual maturity (*L*_*m*_) was determined using the following equation of log (*L*_*m*_) = ˗0.1189 + 0.9157 × log (Lmax) [29]; where *L*_*m*_ = size at first sexual maturity in TL, Lmax = maximum length (TL) of *A. testudineus* in the present study. Furthermore, *L*_*m*_ for water bodies worldwide was estimated using the maximum length of *A. testudineus* retrieved from available literature in the FishBase.

### 2.8 Morphometric and meristic measurements

**Fig. 2:**
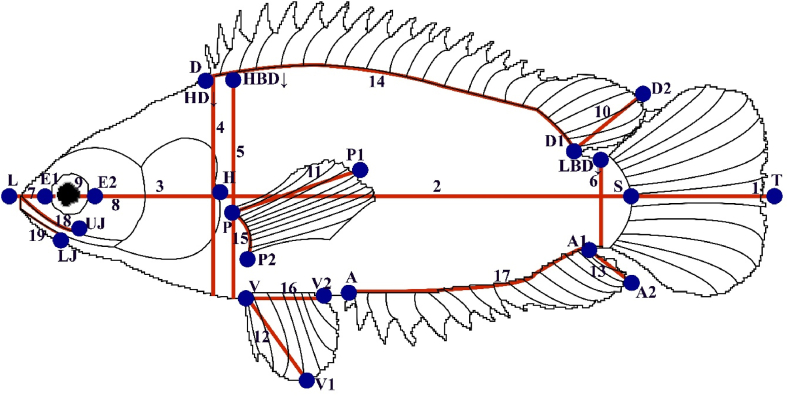
The Position of the 16 landmark points in *A. testudineus*: 1) anterior tip of the upper jaw, 2) point indication of eye commencing, 3) forehead (ending of the frontal bone), 4) the origin of the first dorsal fin,5) origin of dorsal soft rays, 6) end of the dorsal fin, 7) origin of caudal fin (dorsal origin), 8) midpoint of the caudal fin, 9) origin of caudal fin (ventral origin), 10) end of the anal fin, 11) origin of anal soft rays, 12) the origin of the anal fin, 13) origin of the pelvic fin, 14) down point indication of the operculum, 15) origin of pectoral fin, and, 16) end of pectoral fin.

**Fig. 3:**
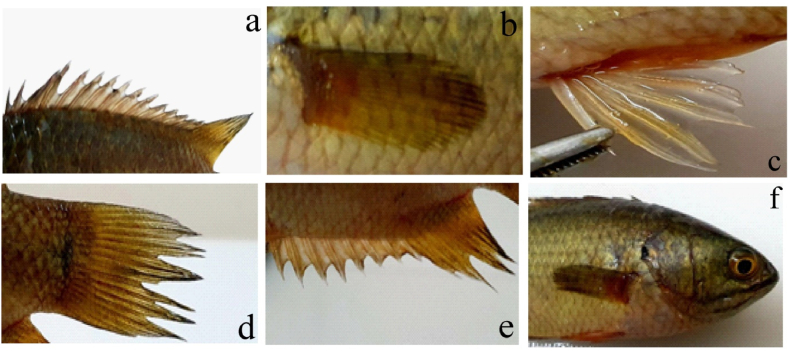
Different fins of *A. testudineus* such as (a) Dorsal, (b) Pectoral, (c) Pelvic, (d) Caudal, (e) Anal, (f) Blackspot and lateral line.

The samples were measured for 20 morphometric characters (Fig. 2) and 12 meristic characters (Fig. 3). Morphometric variables include - Total length (TL), Standard length (SL), Head length (HL), Head depth (HD↓), Head width (HW), Highest body depth (HBD↓), Lowest body depth (LBD↓), Pre-orbital length (LE1), Post-orbital length (HE2), Eye diameter (E1E2), Dorsal fin length (D1D2), Pectoral fin length (PP1), Pelvic fin length (VV1), Anal fin length (A1A2), Base length of dorsal fin (DD1), Base length of pectoral fin (PP2), Base length of pelvic fin (VV2), Base length of anal fin (AA1), Upper jaw length (UJL), and Lower jaw length (LJL) for *A. testudineus*.

Besides, for the meristic characters analysis, 12 characters were taken into consideration for determining any sort of significant differences. The characters counted were: no. of branchiostegal rays (BSR), no. of dorsal fin rays (spine, DFS), no. of dorsal fin rays (soft, DFR), no. of pelvic fin rays (soft, PvFR), no. of pectoral fin rays (spine, PcFS), no. of pectoral fin rays (soft, PcFR), no. of anal fin rays (spine, AFS), no. of anal fin rays (soft, AFR), no. of caudal fin rays (CFR), no. of scale on lateral line (SoLL), no. of scale above lateral line (SaLL), no. of the scale below the lateral line (SbLL) for *A. testudineus*.

### 2.9 Statistical analysis

To normalize the dataset for multidimensional analysis, the size-dependent morphometric characteristics were log-transformed, and each was connected with fish standard length to establish the association between standard length and each morphometric attribute. After establishing the relationship between standard length and morphometric variables, the log-transformed variables were corrected to remove the association of the variables with size. This was accomplished by using the allometric scheme: M_adj_ = M × (L_s_/L_0_) ^b^ [30], where M is the original measurement, M_adj_ is the size-adjusted measurement, L_0_ is the standard length, L_s_ is the mean of standard length, and parameter b is estimated for each character from experimental data as the slope of the regression of log M on log L_0_ using all fish in all populations. PCA was used to reduce redundancy among the morphometric variables and eliminate redundant autonomous variables for population differentiation and cluster heatmap to investigate the phenotypic relations among populations [31,32]. The Statistical Package for Social Sciences (SPSS) version 25.0 was used for statistical analyses at 5% (p < 0.05) and Software R version 3.6.2 (R Core Team, Vienna, Austria) for performing PCA and cluster heatmap analyses. The Wilks' lambda (λ) test was used to estimate the difference between and among the entire populations, and the Kruskal–Wallis test was used to compare means between the groups. In this study, the Wilcoxon sign-ranked test was employed to compare the mean relative weight (*W*_*R*_) with 100 [33].

## 3 Results

### 3.1 Descriptive statistics and length frequency distribution (LFD)

**Fig. 4:**
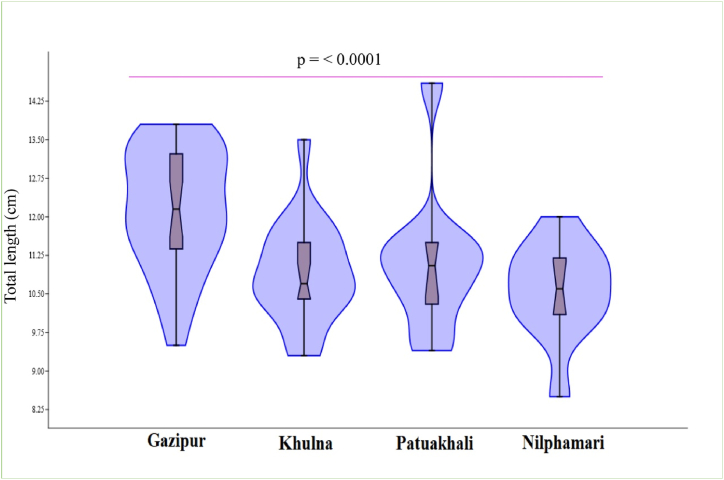
Length frequency distribution of *A. testudineus* samples collected from a different region of Bangladesh.

A total of 120 indigenous *A. testudineus* specimens were collected from Belai *beel* (Gazipur district), Floodplain Dimla (Nilphamari district), the Canal system (Patuakhali district), and Basurabad *beel* (Khulna district) during the study period. Table 1 shows the length and weight ranges and means with standard deviation for specimens obtained from four locations. The average maximum length for *A. testudineus* was caught from Gazipur followed by Patuakhali, Khulna, and Nilphamari, respectively (Table 1). Besides, the average weight was higher in Gazipur, followed by Khulna, Patuakhali, and Nilphamari, respectively (Table 1). The average lengths and weights differ significantly (p˂0.0001). The populations of Gazipur, Nilphamari, Patuakhali, and Khulna were comparable in length. Among all the samples caught, the length class 10.0–11.0 (cm) was dominant except for the samples from Gazipur (Fig. 4).

**Table 1:** Descriptive statistics of climbing perch *A. testudineus* samples from four different regions of Bangladesh.

Population	Sample (n)	Total length		Body weight	
		Length (cm)	Mean ± SD	Weight (gm)	Mean ± SD
Gazipur	30	9.5–13.8	12.15 ± 1.17	16.38–53.21	35.20 ± 10.56
Nilphamari	30	8.50–12.0	10.57 ± 0.94	10.0–29.0	21.0 ± 5.46
Patuakhali	30	9.40–14.60	11.12 ± 1.42	16.82–45.88	25.88 ± 8.30
Khulna	30	9.3–13.5	10.92 ± 0.99	16.25–49.49	27.13 ± 7.65

### 3.2 Length-length relationships (LLRs)

The length-length relationships between TL and SL for *A. testudineus* are presented in Table 2. All LLRs were highly significant (p < 0.001) with the most coefficients of determination values. In this study, the computed allometric coefficient (b) of TL vs. SL shows a positive allometric growth pattern in all the sampling zones.

**Table 2:** Descriptive statistics and estimated parameters of the length-length relationships of *A. testudineus*.

Source	Equation	a ±SE	b ± SE	95% CL p	95% CL q	r^2^	GT
Gazipur	TL = *a* + *b* × SL	1.073 ± 0.418	1.137 ± 0.043	0.216 to 1.931	1.049 to 1.224	0.962	A^+^
Nilphamari	TL = *a* + *b* × SL	1.658 ± 0.493	1.054 ± 0.058	0.543 to 2.774	0.923 to 1.186	0.971	A^+^
Patuakhali	TL = *a* + *b* × SL	1.007 ± 0.200	1.124 ± 0.022	0.546 to 1.469	1.073 to 1.174	0.996	A^+^
Khulna	TL = *a* + *b* × SL	0.509 ± 0.341	1.179 ± 0.038	−0.211 to 1.229	1.098 to 1.260	0.982	A^+^

*a*, intercept; *b*, slope; CL, confidence limit for mean values; GT, growth type; r^2^.

coefficient of determination; A^+^.

positive allometry.

### 3.3 Length-weight relationship (LWRs)

A descriptive statistic and estimated parameters of the length and weight of *A. testudineus* are presented in Table 3. All relationships were highly significant (p < 0.001) with r^2^ values of the species. In the present study, the calculated growth coefficient of the b value of LWRs ranged from 2.377 in Patuakhali to 3.207 in Gazipur, and the coefficients of determination (r^2^) ranged from 0.954 in Patuakhali to 0.976 in Khulna. The LWRs of *A. testudineus* indicated positive allometric growth in the Gazipur and Nilphamari districts (b > 3.00) and negative allometric growth in the Patuakhali and Khulna districts (b < 3.00).

**Table 3:** Descriptive statistics and estimated parameters of the length-weight relationships of *A. testudineus*.

Region	Equation	a ±SE	b ± SE	95% CL a	95% CL b	r^2^	GT
Gazipur	BW = a × TL^b^	−1.947 ± 0.115	3.207 ± 0.106	−1.712 to −2.183	2.990 to 3.426	0.97	A^+^
Nilphamari	BW = a × TL^b^	−1.926 ± 0.181	3.161 ± 0.177	−2.336 to −1.517	2.761 to 3.562	0.973	A^+^
Patuakhali	BW = a × TL^b^	−1.085 ± 0.193	2.377 ± 0.185	−1.529 to −0.638	1.949 to 2.804	0.954	A^-^
Khulna	BW = a × TL^b^	−1.630 ± 0.116	2.942 ± 0.112	−1.875 to −1.387	2.707 to 3.177	0.976	A^-^

*a*, intercept; *b*, slope; CL, confidence limit for mean values; GT, growth type; r2.

coefficient of determination; A+.

positive allometry; A-.

negative allometry.

### 3.4 Condition factors

#### 3.4.1 Allometric condition factor (K_A_)

Patuakhali had the greatest average calculated allometric condition factor (*K*_*A*_) for *A. testudineus*, followed by Khulna, Nilphamari, and Gazipur (Table 4). In Gazipur, there was a significant connection between TL vs*. K*_*A*_ and BW vs*. K*_*A*_, according to Spearman rank-correlation tests, but there was no significant relationship between TL vs*. K*_*A*_ and BW vs*. K*_*A*_ in Nilphamari. Furthermore, in Patuakhali, TL vs*. K*_*A*_ showed an insignificant relationship, whereas BW vs*. K*_*A*_ showed a significant relationship. In Khulna, an insignificant correlation was found between TL vs*. K*_*A*_, while BW vs*. K*_*A*_ was significantly associated (Table 5).

**Table 4:** Descriptive statistics and estimated parameters Condition Factors (*K*_*A*_, *K*_*F*_, *W*_*R*_) of *A. testudineus.*

Species	Condition Factor	Min	Max	Mean ± SD	95% CL
Gazipur	*K*_*A*_	0.0101	0.0126	0.0112 ± 0.0006	0.0110–0.0115
	*K*_*F*_	1.7	2.16	1.89 ± 0.11	1.86–1.94
	*W*_*R*_	90.39	112.538	100.25 ± 5.66	98.14–102.36
Nilphamari	*K*_*A*_	0.011	0.0128	0.0119 ± 0.0005	0.0115–0.0123
	*K*_*F*_	1.6	1.89	1.735 ± 0.09	1.68–1.80
	*W*_*R*_	93.64	108.98	101.336 ± 4.95	98.01–104.67
Patuakhali	*K*_*A*_	0.071	0.088	0.082 ± 0.005	0.0783–0.086
	*K*_*F*_	1.47	2.03	1.85 ± 0.170	1.73–1.97
	*W*_*R*_	85.46	106.301	98.50 ± 6.025	94.19–102.81
Khulna	*K*_*A*_	0.0218	0.0253	0.0235 ± 0.0009	0.0231–0.0240
	*K*_*F*_	1.89	2.2	2.04 ± 0.08	1.99–2.08
	*W*_*R*_	93.01	108.15	100.422 ± 4.129	98.432–102.413

*K*_*A*_, allometric condition factor; *K*_*F*_, Fulton's condition factor; *W*_*R*_, relative weight; Min, minimum; Max, maximum; SD, standard deviation; CL, confidence limit for mean values.

**Table 5:** Relationships of condition factors with total length and body weight of *A. testudineus*.

Species	N	Relationship	r_s_ Value	P values	Significance
Gazipur	30	TL *vs. K*_*A*_	0.4661	0.009	*
		TL *vs. K*_*F*_	0.684	3.052	ns
		TL *vs. W*_*R*_	0.4661	0.009	*
		BW *vs. K*_*A*_	0.581	0.001	*
		BW *vs. K*_*F*_	0.7802	3.699	ns
		BW *vs. W*_*R*_	0.581	0.001	*
Nilphamari	30	TL *vs.* K_A_	0.3514	0.289	ns
		TL *vs. K*_*F*_	0.6083	0.047	*
		TL *vs. W*_*R*_	0.3514	0.289	ns
		BW *vs. K*_*A*_	0.3957	0.228	ns
		BW *vs. K*_*F*_	0.6587	0.027	*
		BW *vs. W*_*R*_	0.3957	0.228	ns
Patuakhali	30	TL *vs. K*_*A*_	0.4988	0.142	ns
		TL *vs. K*_*F*_	0.0890	0.807	ns
		TL *vs. W*_*R*_	0.4988	0.142	ns
		BW *vs. K*_*A*_	0.5705	0.085	*
		BW *vs. K*_*F*_	0.1567	0.665	ns
		BW *vs. W*_*R*_	0.5705	0.085	*
Khulna	30	TL *vs. K*_*A*_	0.3285	0.170	ns
		TL *vs. K*_*F*_	0.2283	0.347	ns
		TL *vs. W*_*R*_	0.3285	0.170	ns
		BW *vs. K*_*A*_	0.4932	0.032	*
		BW *vs. K*_*F*_	0.3970	0.092	*
		BW *vs. W*_*R*_	0.4932	0.032	*

TL, total length; BW, body weight; *K*_*A*_, allometric condition factor; *K*_*F*_; Fulton's condition factor; *W*_*R*_, relative weight; *r*_*S*_, Spearman rank-correlation values; *p*, shows the level of significance; *ns*, not significant; * significant.

#### 3.4.2 Fulton's condition factor (K_F_)

The average computed Fulton's condition (*K*_*F*_) for *A. testudineus* was highest in Khulna, then highest in Gazipur, Patuakhali, and Nilphamari (Table 4). No significant relation was found between TL vs*. K*_*F*_ and BW vs*. K*_*F*_ in Gazipur, but they were significantly correlated with one another in Nilphamari, according to Spearman rank-correlation analyses. In Patuakhali, it was found that there was no difference between TL vs*. K*_*F*_ and BW vs*. K*_*F*_. In Khulna, a significant connection between BW vs*. K*_*F*_ was discovered, whereas TL vs*. K*_*F*_ showed an insignificant relationship (Table 5).

#### 3.4.3 Relative weight (W_R_)

The average calculated relative weight (*W*_*R*_) for *A. testudineus* was found highest in Nilphamari, followed by Khulna, Gazipur, and Patuakhali (Table 4). According to Spearman rank-correlation tests, there was a significant relationship between TL vs*. W*_*R*_ and BW vs. *W*_*R*_ in Gazipur, whereas no significant relationship between TL vs*. W*_*R*_, and BW vs*. W*_*R*_ in Nilphamari. Furthermore, a significant relationship was distinguished between BW vs*. W*_*R*_ while no significant relationship was observed between TL vs*. W*_*R*_ in Patuakhali. In addition, a significant relationship between BW vs*. W*_*R*_ was observed, while an insignificant relationship was found between TL vs*. W*_*R*_ in Khulna (Table 5).

### 3.5 Form factor (*a*_*3.0*_)

**Fig. 5:**
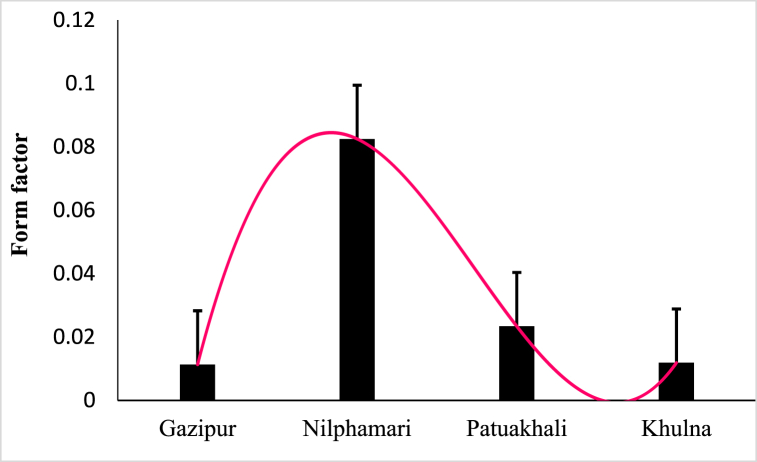
Form factor (*a*_*3.0*_) of *A. testudineus* from four different water habitats of Bangladesh.

The calculated form factor (*a*_*3.0*_) values for all the habitats showed a short and deep body shape. The researchers also used available data to compute the *a*_*3.0*_ of *A. testudineus* from several aquatic water bodies around the world, which were compared to the current study (Fig. 5 and Table 6).

**Table 6:** The calculated form factor (*a*_*3.0*_) and size at first sexual maturity (*L*_*m*_) of *A. testudineus* in different water bodies worldwide.

Water body	L_max_ (cm)	(*a*_*3.0*_)	L_m_ (cm)	95% CL of Lm	Reference
Belai *beel*, Gazipur	13.8	0.0113	8.42	7.237–7.733	Present study
Floodplain, Nilphamari	12.0	0.0825	7.40	6.228–6.950	Present study
Canal system, Patuakhali	11.4	0.0234	8.86	6.324–7.474	Present study
Basurabad *beel*, Khulna	13.5	0.0110	8.24	6.513–7.056	Present study
Tetulia river, BD	16.1	0.0161	9.26	6.67–12.87	Hossain, et al., 2015
Kausalyaganga, India	17.5	0.0859	10.00	7.17–13.96	Kumar et al., 2013
West Bengal, India	17.0	0.0077	9.74	6.99–13.57	Ziauddin et al., 2016
Chi river, Thailand	16.5	0.0167	9.47	6.81–13.18	(Satrawaha and Pilasamorn, 2009)

Sex was not considered; BD, Bangladesh.

### 3.6 Size at first sexual maturity (L_m_)

The maximum *L*_*m*_ for *A. testudineus* was estimated as 8.42 (∼ 8.4) in Gazipur followed by Patuakhali, 8.86 (∼ 8.9), Khulna, 8.24 (∼ 8.2), and 7.40 (∼ 7.4) in Nilphamari. Additionally, data for *A. testudineus* accessible from various publications were calculated for the comparative studies of *L*_*m*_ (Table 6).

### 3.7 Morphometric and meristic measurements

#### 3.7.1 Morphometric characteristics

**Fig. 6:**
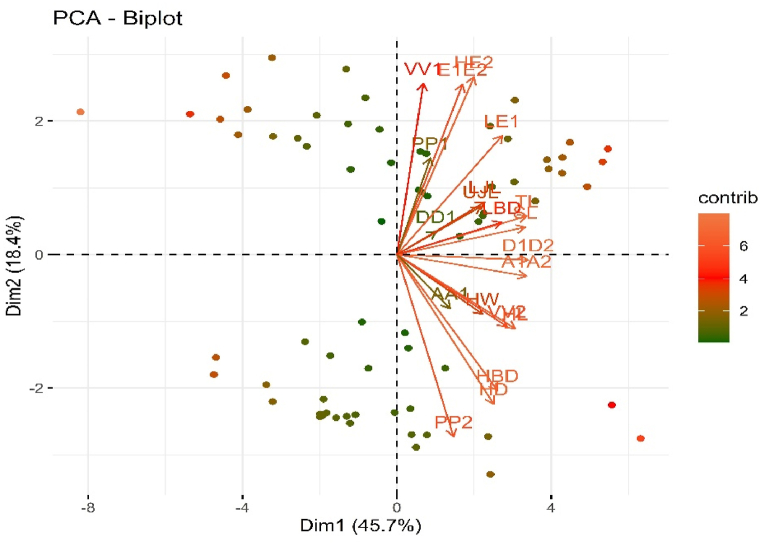
The bi-plots of PCA demonstrating dimension results denote the loadings of every character for morphometric of *A. testudineus* samples collected from a different region of Bangladesh.

Most of the morphometric characters of *A. testudineus* showed a significant difference (p < 0.05) among all the features except the length of the dorsal fin base among different stocks of *A. testudineus* (Table 7). Those significant characters were then used for multivariate analysis (PCA, and cluster heatmap analysis). The contribution of a variable to PCA was examined for determining which morphometric characters generate extreme variance among the populations (Fig. 6). In PCA, 20 morphometric dimensions extracted five factors with eigenvalues >1, elucidating 85.361% of the total change in TL, SL, HL, LBD, LE1, D1D2, A1A2, and VV2 (Table 8).

**Table 7:** Morphometric characters showing significant and insignificant differences among different stocks of indigenous koi *(A. testudineus)* based on Wilks' Lambda and Kruskal-Wallis (H, Chi-Square) test.

Morphometrics	Wilks' Lambda	*P* value	Chi-Square	*P* value
TW	0.722	< 0.0001	19.531	< 0.0001
TL	0.740	< 0.0001	19.431	< 0.0001
SL	0.780	0.001	17.024	0.001
HL	0.735	< 0.0001	19.129	< 0.0001
HD↓	0.452	< 0.0001	29.977	< 0.0001
HW	0.800	0.002	11.688	0.009
HBD↓	0.152	< 0.0001	42.329	< 0.0001
LBD↓	0.094	< 0.0001	45.848	< 0.0001
LE1	0.290	< 0.0001	48.371	< 0.0001
HE2	0.260	< 0.0001	51.130	< 0.0001
E1E2	0.208	< 0.0001	59.482	< 0.0001
D1D2	0.717	< 0.0001	20.634	< 0.0001
PP1	0.650	< 0.0001	23.689	< 0.0001
VV1	0.478	< 0.0001	36.174	< 0.0001
A1A2	0.651	< 0.0001	23.343	< 0.0001
DD1	0.969	0.556	25.331	< 0.0001
PP2	0.429	< 0.0001	41.728	< 0.0001
VV2	0.211	< 0.0001	37.804	< 0.0001
AA1	0.759	< 0.0001	21.303	< 0.0001
UJL	0.502	< 0.0001	34.081	< 0.0001
LJL	0.083	< 0.0001	62.316	< 0.0001

Values are significantly different at (p < 0.05).

**Table 8:** Eigenvalues, percentage of variance, and percentage of cumulative variance for the five factors of *A. testudineus* samples collected from a different region of Bangladesh.

Component	Eigenvalues	% of Variance	Cumulative %
1	9.131	45.655	45.655
2	3.687	18.436	64.091
3	2.318	11.588	75.679
4	1.059	5.294	80.973
5	0.878	4.388	85.361

**Fig. 7:**
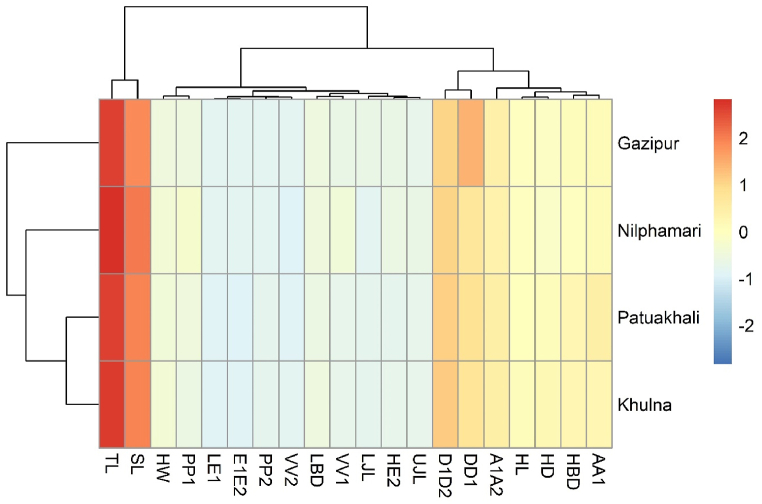
Cluster heatmap analyses of morphometric characteristics of *A. testudineus* samples collected from a different region of Bangladesh.

The first principal component (PC1) defined 45.655% of the total variation, the second principal component (PC2) described 18.436%, PC3 described 11.588%, PC4 described 5.294% and PC5 described 4.388% (Table 9). The most noteworthy loadings on PC1 were TL, SL, HL, HD↓, HW, HBD↓, LBD↓, LE1, HE2, E1E2, D1D2, A1A2, PP2, VV2, AA1, UJL, LJL, on PC2; HD↓, HBD↓, PP2, and on PC3; PP1, VV1 were significant and others were PC4 and PC5, which were not significant (Table 9). The morphometric traits with an eigenvalue >1 were involved, and others were omitted in this analysis. It can be mentioned which factor loading >0.30 is regarded as significant, >0.40 is regarded as more significant, and >0.50 or above is regarded as highly significant [34]. The factors are considered important with loadings larger than 0.5 in this study. The cluster heatmap revealed three major clades: the first includes populations from the Nilphamari district, the second includes populations from the Patuakhali and Khulna districts, and the third includes populations from the Gazipur district (Fig. 7).

**Table 9:** Results of factors extraction in PCA after varimax normalized rotation of *A. testudineus* samples collected from a different region of Bangladesh.

Morphometrics	PC 1 (45.655%)	PC 2 (18.436%)	PC 3 (11.588%)	PC 4 (5.294%)	PC 5 (4.388%)
TL	**0.953**	−0.016	0.216	−0.096	0.011
SL	**0.944**	0.016	0.219	−0.096	0.001
HL	**0.880**	0.329	0.134	−0.107	−0.021
HD↓	**0.763**	**0.570**	−0.019	−0.042	−0.013
HW	**0.701**	0.289	0.276	−0.282	−0.051
HBD↓	**0.799**	**0.516**	0.031	0.054	−0.039
LBD↓	**0.810**	−0.020	0.273	−0.161	0.077
LE1	**0.840**	−0.424	−0.142	0.003	0.021
HE2	**0.757**	−0.523	0.094	−0.169	−0.099
E1E2	**0.677**	−0.584	−0.102	−0.123	−0.197
D1D2	**0.955**	0.101	0.138	−0.053	0.002
PP1	0.444	−0.208	**0.804**	0.126	0.076
VV1	0.429	−0.518	**0.621**	−0.011	0.030
A1A2	**0.947**	0.145	0.085	0.003	0.052
DD1	0.158	−0.100	−0.113	0.081	0.919
PP2	**0.517**	**0.731**	0.060	0.044	0.066
VV2	**0.790**	0.261	−0.499	−0.012	0.031
AA1	**0.548**	0.256	0.161	0.452	−0.088
UJL	**0.801**	−0.056	−0.048	0.079	−0.191
LJL	**0.766**	−0.208	−0.488	0.123	−0.130

The loadings are significant at p > 0.05. The utmost significant morphometric characters underwritten from PC1 to PC5 are marked as the boldface.

#### 3.7.2 Meristic characters

The majority of the meristic characters of *A. testudineus* showed significant differences (p < 0.05) among all the features except no. of dorsal fin rays (spine) and no. of dorsal fin rays (soft) showed no significant difference (Table 10).

**Table 10:** Meristic characters showing significant and insignificant differences among different stocks of *A. testudineus* based on Wilks' Lambda and Kruskal-Wallis (H, Chi-Square) test.

Meristics	Wilks' Lambda	*P* value	Chi-Square	*P* value
BSR	a	–	69.000	< 0.0001
DFS	0.909	0.096	7.051	0.070
DFR	0.955	0.382	3.363	0.339
PvFR	0.342	< 0.0001	54.350	< 0.0001
PcFS	a	–	69.000	< 0.0001
PcFR	0.076	< 0.0001	64.397	< 0.0001
AFS	0.736	< 0.0001	21.004	< 0.0001
AFR	0.758	< 0.0001	44.749	< 0.0001
CFR	0.117	< 0.0001	58.911	< 0.0001
SoLL	0.141	< 0.0001	39.718	< 0.0001
SaLL	0.111	< 0.0001	60.548	< 0.0001
SbLL	0.055	< 0.0001	63.272	< 0.0001

a cannot be computed because this variable is constant in each population and significant (p < 0.01).

## 4 Discussion

The length-frequency distribution shows the structure of fish populations, which is useful for fisheries management. In the current study, it was not possible to sample individuals of *A. testudineus* below 8.5 cm TL due to a bias in the collection of fishing gear or because fishermen did not go where the smaller fish were, or possibly due to a scarcity of small fish on the fishing grounds. The minimum size (TL) of *A. testudineus* was 8.5 cm in Nilphamari and the maximum size (TL) was 14.4 cm in Patuakhali, respectively, which is lower than the maximum recorded value of 25.0 cm [35], 16.50 cm [36], and 16.10 cm [16]. Maximum length is used in fisheries resource planning and management to determine the asymptotic length and growth coefficient [37,38]. The allometric or isometric growth rate is specified by the parameter b value, and if it remains stable and assumes equal to or greater than 3.0, the individual's shape and ontogenetic growth are unaffected. The calculated growth coefficient of the b value of LWRs in this study varied from 2.377 in Patuakhali to 3.207 in Gazipur, which was within the usual range of b values for fishes (2.50–3.50) [11]. Whereas the b values being close to 3 signify that fish grow isometrically, and values > 3 indicate positive allometry, and <3 indicate negative allometry [27].

In this study, the LWRs of *A. testudineus* indicated positive allometric growth in the Gazipur and Nilphamari districts (b > 3.00) and negative allometric growth in the Patuakhali and Khulna districts (b < 3.00). Other research has revealed that *A. testudineus* has negative allometric growth b = 2.86 in Agushan marsh of Philippines [39]; b = 2.91 from the Tetulia river in Bangladesh [16], and b = 2.77 from ponds in Kausalyaganga of India [18]. Such discrepancies can be caused by changes in observed length class, preservation technique, stock health, stomach fullness, gonadal maturity, gender, season, or geographic location [19], which were not taken into account in our investigation.

A fish's condition factor (*K*) represents physical and biological conditions and variations as a result of interactions between feeding conditions, parasite infections, and physiological parameters [26]. This also reflects changes in food reserves as well as markers of overall fish health. As a result, knowledge of condition factors is important for culture system management since it informs the producer about the exact conditions in which organisms are growing [40]. Besides, the condition factor is an index that represents interactions between biotic and abiotic elements of the fish's physiological condition to determine the population's well-being during various life cycle stages [11]. Moreover, condition factor decrease during low temperatures, low food availability, spawning season approaches, after spawning, particularly in females, and a second increase after spawning [26]. In the current study, the condition factor (*K*_*F*_) for *A. testudineus* was found minimum of 1.89 in Gazipur and a maximum of 2.04 in Khulna districts, respectively. The *K*_*F*_ value > 1 indicates perfect condition whereas, <1 reflects that the well-being of the fish is not in good condition [11,27]. So, *K*_*F*_ is the best condition index for assessing well-being among other condition factors which were significantly correlated with TL and BW. According to Spearman rank-correlation analyses, no substantial relationships were observed between BW vs*. K*_*F*_ in Gazipur, but in Nilphamari. In Patuakhali, there was no distinction between TL vs*. K*_*F*_ and BW vs*. K*_*F*_. In Khulna, it was found that there was a significant connection between BW vs*. K*_*F*_, but not between TL vs*. K*_*F.*_ The *W*_*R*_ is useful in determining population health and fitness, as well as ecosystem disturbances [14]. A fish with a higher relative weight has more energy reserves available for regular activities, development, and reproduction [41]. The relative weight of less than 80 is considered very slim, 90 is considered normal, and greater than 100 is considered obese [14,42]. Relative weight is one of several common measures of condition used in fisheries assessment and management [42]. It is ideal that standard weight equations encompass a species' whole geographical range and be used for comparison rather than management aims [41]. The *W*_*R*_ values for an individual, size group, or population when >100 suggest such low prey availability or high predatory density; whereas values < 100 indicate a prey surplus or low predatory density [13,14,43]. The *W*_*R*_ values for *A. testudineus* in this study were close to 100, indicating that the species is bigger and fatter, with good health and reproduction. The present finding conforms with the findings of [13,41,43,44]. In Gazipur, TL vs*. W*_*R*_ and BW vs. *W*_*R*_ revealed a significant relationship according to Spearman rank-correlation analyses. Additionally, a significant relationship between BW vs*. W*_*R*_ was identified in Patuakhali, as well as a significant connection in Khulna. However, the current study was to carry out a comprehensive description of *W*_*R*_ for the *A. testudineus* in a different region of Bangladesh. The Spearman rank-correlation test of relative weight *W*_*R*_ was substantially linked with TL and BW, indicating that *W*_*R*_ is the best condition metric for measuring the well-being of *A. testudineus* in the studied habitats and surrounding ecosystems. The present finding is in disconformity with the previous findings of [45].

The form factor can be used to see if a population's or organism's body shape is considerably different from that of others [11]. The form factors of *A. testudineus* were estimated minimum of 0.0110 in Khulna and a maximum of 0.0825 in Nilphamari, respectively suggesting an elongated body shape typical of many riverine fishes. The estimated *a*_*3.0*_ was 0.021 for *A. testudineus* in Gajner *beel* which fairly supports the present study [45].

The first sexual maturity size can be used to calculate the minimum capture size and assess the stock [46]. Size at first sexual maturity (*L*_*m*_) was estimated minimum of 7.40 (∼ 7.4) cm TL in Nilphamari and a maximum of 8.86 (∼ 8.9) cm TL in Patuakhali, correspondingly. The *L*_*m*_ was calculated as 8.41 (∼8.40) cm TL in the Gajner *beel* [45] which supports the present findings and constitutes a baseline in different habitats for future studies of environmental factors affecting *L*_*m*_ and spawning season.

There were highly significant morphological variations reported among *A. testudineus* populations from all analyzed sites in the current study. Almost all morphometric parameters exhibited substantial changes between them, except for the length of the dorsal fin base, which was not significantly different between different *A. testudineus* populations. These considerable variances can be attributed to the environment, ecology, human activity, and genetic variety. Related research was conducted on both non-reproductive and reproductive *Rutilus frisii kutum* in the southwest Caspian Sea [47], two genetically distinct populations of *Lethenteron reissneri* from Hokkaido and Honshu islands, Japan [48], Mahseer (*Tor putitora*) from a Himalayan foothill river's spawning ground, and Pakistan [49] where the majority of the morphometric characters display considerable variation. There has also been some research done on morphometric measurements and meristic counts on various fish species, with variances observed due to geography, ecology, and human activity [50]. However, because the stocks were gathered from different areas and may have descended from distinct ancestors, morphometric discrepancies between them are to be expected [51], and they may be able to adapt. Fish are highly sensitive to changes in their environment (such as food supply and temperature), and they adapt their morphometric traits accordingly [9,52]. Fish show more morphological variation within and between species than any other vertebrate, as well as being more sensitive to environmental-induced morphological alterations [53]. Multivariate morphometric character analysis, on the other hand, is a quick and easy way to assess a large number of fish's discrimination [54].

Multivariate analysis was performed which includes PCA and cluster heatmap to find discrepancies among the stocks. In terms of morphometric features, PCA and cluster heatmap distinguished the Gazipur district population from the Nilphamari district, Khulna district, and Patuakhali district populations in terms of TL, SL, HL, LBD, LE1, D1D2, A1A2, and VV2, which could be linked to physical distance, as it is widely known that geographic separation causes morphological alterations [55]. When a species' distribution is largely consistent over a range, the equilibrium between gene flow and population differentiation processes such as genetic drift or differential selection can lead to inclines, in which genetic divergence increases as geographic distance increases [50]. The distance between the rivers, the diverse geographical locations, and the environmental limits that each population experiences can all contribute to population differences. The location, environmental, and ecological variables all affect population morphometric discrimination [9,56]. In the current study, the size effect for morphometric data was successfully recovered using allometric transformation, demonstrating that morphological variations of *A. testudineus* within a distinct region of Bangladesh could be associated with variances in head and body shape. As a result, changes in food quantity, as well as abiotic factors such as tidal fluctuation, salinity, turbidity, dissolved oxygen, and other factors may be responsible for the separation of the four *A. testudineus* populations.

The meristic variables are utilized in taxonomic categorization and are used to explain stock recognition [57]. The meristic counts of all populations vary from 4 to 5 for branchiostegal rays, 15–18 for dorsal fin rays (spine), 8–9 for dorsal fin rays (soft), 14–18 for pelvic fin rays (soft), 1 pectoral fin rays (spine), 5–9 for pectoral fin rays (soft), 10–12 for anal fin rays (spine), 9–11 for anal fin rays (soft), 15–20 for caudal fin rays, 26–31 for scale on lateral line, 3–6 scale above the lateral line, 4–12 for scale on the below the lateral line. This outcome is comparable to the taxonomic formula of several studies [1,2].

The meristic characters showed a significant difference among the samples from a different region of Bangladesh, but the two-character namely no. of dorsal fin rays (spine) and no. of dorsal fin rays (soft) showed no significant difference among different stocks of *A. testudineus*. A related study was executed on non-reproductive and reproductive kutum females (*Rutilus frisii kutum*) in the southwest Caspian sea [47] and no discernible distinction between the meristic characters was found. However, meristic counts of endangered carp, *Labeo calbasu*, from two isolated river stocks, the Jamuna and the Halda, and a hatchery differ [51]. Meristic characteristics within a species remain constant [49]. Significant variations in the meristic and biological characteristics of European anchovy *Engraulisen crasicolus* collected from the Eastern, Western, Marmara, and Aegean seas were reported [58]. The temperature, radiation, salinity, and dissolved oxygen are all factors that have an impact on meristic properties [59]. The geographical differences invariably result in dramatic changes in environmental conditions. The fact that *A. testudineus* is found in different geographical regions and is exposed to varied environmental conditions explains the majority of the meristic variants.

## 5 Conclusion

The current findings provide comprehensive evidence on intraspecific phenotypic variations in climbing perch *A. testudineus* populations for habitat adaptations using multi-linear models throughout Bangladeshi water bodies. The findings of this study can be used by fishery biologists, conservationists, and managers to develop management and fishery regulations for the long-term conservation of the surviving stocks. Furthermore, the landmark-based technique can be used to examine the variation of stocks within a species, as demonstrated by the clear variation in the wild stocks of *A. testudineus* in Bangladesh's four sampling regions, indicating the need for distinct management approaches to protect the stocks and ensure their long-term viability. However, molecular differentiation, in combination with morphological characteristics, gives valuable information about a species that may be used to develop scientifically sound fisheries management strategies for specific species in specific habitats.

## Author contribution statement

Md. Rased Khan Manon: Performed the experiment, acquisition of data, and wrote the paper; Asraful Alam and Md. Rahamat Ullah: Analyzed and interpreted the data; Md. Belal Hossen and Md. Abu Sufian: Contributed materials and acquisition of data; Mohammad Amzad Hossain: Conception and design of the study; Mohammed Mahbub Iqbal: Critically revising; Md. Arifur Rahman: Conception and design of the study, wrote the paper, final approval of the version, and supervision.

## Funding statement

This research was self-funded.

## Data availability statement

Data will be made available on request.

## Declaration of competing interest

The authors declare that they have no known competing financial interests or personal relationships that could have appeared to influence the work reported in this paper.
